# A Combination of Enhanced Mechanical and Electromagnetic Shielding Properties of Carbon Nanotubes Reinforced Cu-Ni Composite Foams

**DOI:** 10.3390/nano11071772

**Published:** 2021-07-07

**Authors:** Dan Wang, Zhong Wu, Fengxian Li, Xueping Gan, Jingmei Tao, Jianhong Yi, Yichun Liu

**Affiliations:** 1 School of Materials Science and Engineering, Kunming University of Science and Technology, Kunming 650093, China; doromerd@163.com (D.W.); clfxl@kust.edu.cn (F.L.); kiwimaya@126.com (J.T.); yijianhong@kmust.edu.cn (J.Y.); 2 School of Materials Science and Engineering, Tianjin University, Tianjin 300350, China; wuzhong2319@163.com; 3 State Key Laboratory of Powder Metallurgy, Central South University, Changsha 410083, China; ganxueping@csu.edu.cn

**Keywords:** carbon nanotubes, metal matrix composite foam, electrodeposition, compression property, electromagnetic shielding

## Abstract

Carbon nanotubes (CNTs) reinforced double-layered Cu-Ni composite foams (Cu-Ni/CNT foams) were prepared through chemical plating and electrodeposition, for the purpose of combining enhanced mechanical and electromagnetic shielding properties. The microstructure characterization revealed a quite uniform dispersion of the CNTs embedded in the metal layers, even after heat treatments. The property testing showed the compressive strength, energy absorption capacity and electromagnetic shielding effectiveness (SE) of Cu-Ni/CNTs foams were significantly improved, as compared to Cu-Ni foams. The heat treatments of the composite foams resulted in an interdiffusion of the Cu and Ni layers, causing an increase of compressive strength and a slight decrease of average SE. The possible mechanisms of the property evolution are discussed.

## 1. Introduction

Lightweight metal foams can be effectively used in noise reduction, heat insulation, shock absorption, electromagnetic shielding and other aspects [1,2,3]. The metallic foams prepared by the electrodeposition process have the advantages of a complete and continuous pore structure, high electrical conductivity, fast heat dissipation, which can be applied to various practical applications, such as heat dissipation of various electronic equipment, ventilation and the electromagnetic enhancement of light transmission equipment [4,5,6]. In recent years, the application of metal foam has gained much interest. However, the surface binding force of the metal foam is reduced and stress concentration is easily generated around the pores, which leads to a decrease in mechanical properties [7]. Improving the mechanical properties is a key factor for the application of metal foams. According to reports, Ni coatings generally provide metal foam with high strength and good wear and corrosion resistance [8]. Du et al. [9] deposited a thin layer of Ni coating on the pore walls of lotus-shaped porous Cu through an electroplating process, and the compressive strength and energy absorption of porous Cu have increased by about 30–50%. At the same time, metal foams also have certain advantages in electromagnetic shielding. When the electromagnetic shielding efficiency is equivalent, the metal foam material is only 1/3 weight of the traditional ventilated waveguide [10,11]. In order to expand the application range of metal foam, CNTs with high strength and high conductivity are used in metal foam to improve the mechanical properties and electromagnetic shielding performance [12,13,14]. Ji et al. [15] studied the electromagnetic shielding performance of Cu-Ni-CNTs foam composites in the 8–12 GHz frequency band by depositing a layer of CNTs on the Cu-Ni foam framework. The average electromagnetic shielding effectiveness could reach 47.5 dB.

With high mechanical strength, excellent electrical/thermal conductivities, as well as outstanding electromagnetic wave absorbing properties, CNTs are considered as one of the best reinforcement candidates for metal matrix composites [4]. Our previous study has shown an obvious enhancement of both compression and electromagnetic shielding properties with the incorporation of CNTs into the Cu foams [16]. In this paper, Cu-Ni foams with embedded CNTs as the reinforcements were studied, which were prepared by a combination of electroless plating and composite electrodeposition with open-cell melamine foams as the templates. As is well-known, Cu has high microwave reflectivity, while Ni and CNTs have good microwave absorbability, so the Cu-Ni/CNTs composite foams were expected to possess enhanced electromagnetic shielding effectiveness, as well as improved mechanical properties. The structure, properties and possible mechanisms of the composite foams are investigated and discussed in this paper.

## 2. Experimental Section

### 2.1. Preparation of Composite Foams

**Figure 1:**
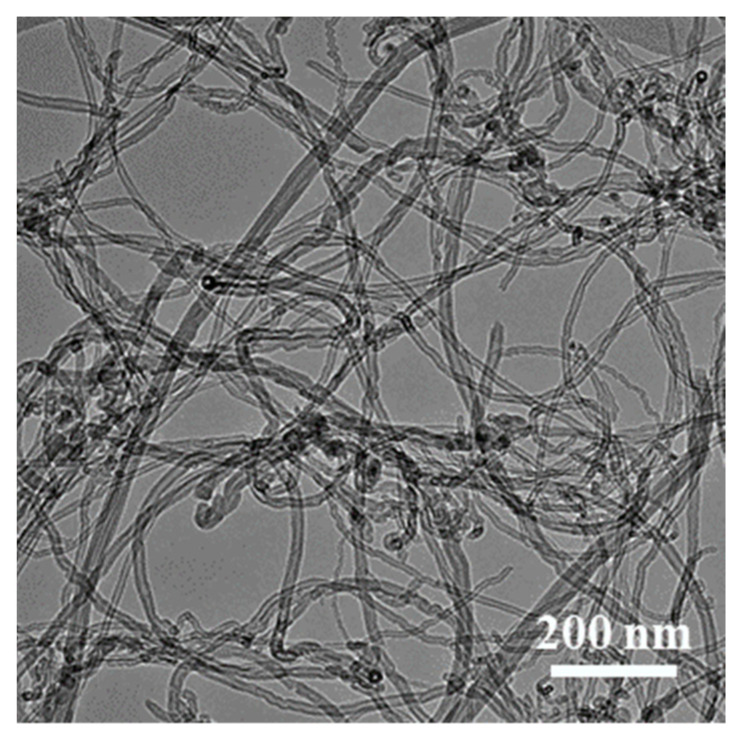
The TEM image of raw CNTs.

The template method was used to prepare open-cell composite foams. The melamine foam (Outlet Technology Co. Ltd., Chengdu, China) with open porosity of about 99.8%, pore diameter of 100–200 μm and wire diameter of 6–9 μm was used as a template. The experiment mainly included the following steps: (1) chemical plating of silver deposits on melamine foams to make it conductive. The melamine foam in the experiment was cut into a cylindrical shape with diameter of 30 mm and height of 5 mm. First, ammonia water was added drop wise to the silver nitrate solution (20 g/L), shaking while dripping, until the initially formed precipitate just dissolved. Then, the melamine foam was immersed in the silver mirror reaction solution. The glucose solution (100 g/L) was dropped into the solution and reacted at 25 °C for 8 min and then taken out, rinsed and dried. (2) Composite electrodeposition of Cu/CNTs layers on the silver-plated foam surface. The electrodeposition solution contained CuSO_4_·5H_2_O (200 g/L), H_2_SO_4_ (60 g/L), polyethylene glycol (0.03 g/L), 2-butyne-1,4-diol (0.2 g/L) and CNTs dispersant (1 g/L). The CNTs dispersant (Chengdu Organic Chemistry Co. Ltd., Chengdu, China) was composed of 10 wt.% of CNTs (inner diameter of 5–20 nm, outer diameter of 30–50 nm and average length of 10 μm, as shown in Figure 1). The copper plate was used as the anode, and the silver-plated foam was used as the cathode. Electrodeposition was conducted for 7 h at a current density of 2 A/dm^2^. After electrodeposition, the foams were rinsed with deionized water and dried at 60 °C for 4 h. (3) Composite electrodeposition of Ni/CNTs layers on the Cu/CNTs foam. Electrodeposition solution contained: NiSO_4_·6H_2_O (80 g/L), NiCl_2_·6H_2_O (80 g/L), saccharin (1 g/L), boric acid (40 g/L), sodium dodecyl sulfate (80 mg/L), 2-butyne-1,4-diol (0.4 g/L), CNTs dispersant (1 g/L). The nickel plate was used as the anode, and Cu/CNTs foam was used as the cathode. Electrodeposition is carried out for 1 h at a current density of 3 A/dm^2^. After electrodeposition, the foams were rinsed with deionized water and dried at 60 °C for 4 h. The same process and steps were used to prepare Cu-Ni foams without CNTs in the solution. (4) The heat treatment process was carried out in a furnace with two stages in a reducing atmosphere (volume ration of H_2_: N_2_ = 1:10). At the first stage, the foam was heated at 650 °C for 1 h to pyrolyze the melamine template. At the second stage, the foam was heated at 800 °C for 1 h. The samples and the related parameters are listed in Table 1.

### 2.2. Characterization

The microstructure of surface and cross sections of the foams were characterized by field emission scanning electron microscopy (FE-SEM, Nova Nano-450, FEI, Hillsboro, OR, USA). An X-ray energy dispersive spectroscope (EDS) equipped in the electron microscope analyzed the composition of the sample. The microstructures of the CNTs and composites were revealed by transmission electron microscopy (TEM, Tecnai G2-TF30 S-Twin, FEI, Hillsboro, OR, USA). The TEM samples were first ground from copper layer to a certain thickness, and then thinned by ion milling method (GATAN PISP 691 type, Gatan, Inc., Pleasanton, CA, USA). As the nickel layer of the sample was relatively thin, the prepared transmission sample was dominated by the copper layer. The analysis of the phase was carried out with X-ray diffraction analyzer (FJ-2000). The surface structure changes of CNTs were characterized by LabRam HR Evolution. The compression properties were tested with a mechanical tester (AG-X-100kN, SHIMADZU, Kyoto, Japan) at a constant rate of 0.1 mm/min at room temperature. The electromagnetic shielding performance was tested by vector network instrument (Key-sight E5071C ENA, Agilent, Santa Clara, CA, USA) in the waveguide mode, and the test frequency band was 8.2–12.4 GHz. The dimensions of the electromagnetic shielding test samples were unified as rectangular parallelepipes with length of 22.9 mm, width of 10.2 mm, and height of 3 mm. The CNTs content of each sample were tested by a C/S analyzer (SDF2-HCS878, Beijing Hifid Technology Co. Ltd., Beijing, China).

## 3. Results and Discussion

### 3.1. Morphology and Composition of the Composite

**Figure 2:**
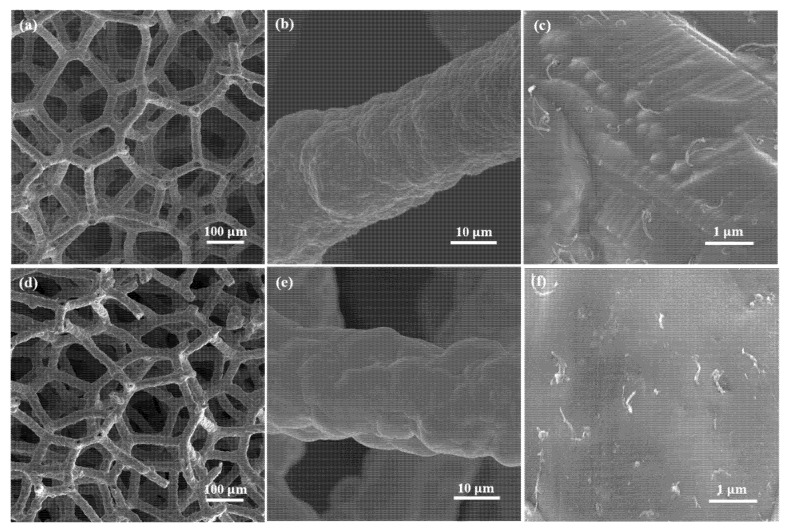
The SEM observation of Cu-Ni/CNTs foams at different magnifications; (**a**–**c**) before heat treatments; (**d**–**f**) after heat treatments.

Figure 2 shows the morphologies of Cu-Ni/CNTs foams at different magnifications before and after heat treatments. The 3D network structure and the dense coating surface of the open-cell composite foams can be seen clearly. The CNTs were uniformly distributed without obvious agglomeration in the foam skeleton. One of the differences as compared to reference [15] was that the CNTs in our work were embedded in the metal matrix rather than being electrophoretically deposited on the surface. It is believed the embedded CNTs provide an effective load transfer and are beneficial to improve the mechanical properties of the composite [17].

**Figure 3:**
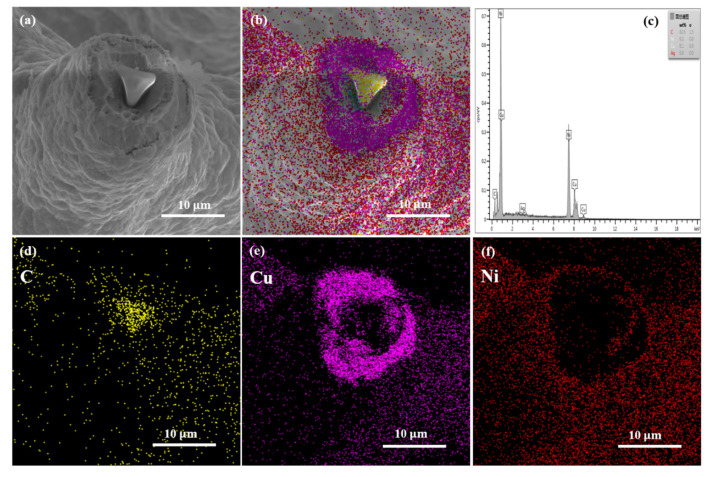
The cross section of Cu-Ni/CNT foams before heat treatment; (**a**) SEM observation; (**b**–**f**) EDS analysis of the cross section.

Figure 3a shows the SEM cross-sectional photograph of Cu-Ni/CNT foam skeleton. It can be seen that the thickness was not relatively uniform at the micrometer scale because the granules of the coatings were inhomogeneous. Figure 3b–f show the EDS analysis of cross section of Cu-Ni/CNT foams. The EDS results illustrate that the electrodeposited foam was composed of a melamine template, the inner Cu/CNT layer and the outer Ni/CNT layer. The Ni coating with a thickness of about 1 µm was tightly bound to the Cu layer. The ratio of Cu layer thickness to Ni layer thickness was approximately 6:1. In this case, the thin nickel coating was uniformly deposited on the walls of Cu foams, with little change of the foam weight and pore size. Furthermore, the outer Ni layer on Cu metal may improve the corrosion resistance.

**Figure 4:**
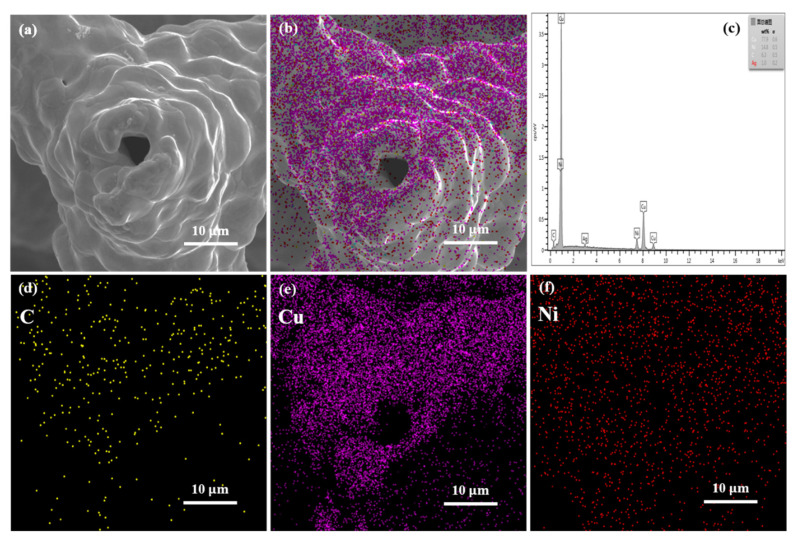
The cross section of Cu-Ni/CNTs foams after heat treatment; (**a**) SEM observation; (**b**–**f**) EDS analysis of the cross section.

Figure 4 shows the cross-sectional observation of the Cu-Ni/CNT foam skeleton after heat treatment. The melamine template has been removed and a pore has been left in the skeleton center. The EDS analysis results in Figure 4b–f show that Cu and Ni atoms both appeared in the inner and outer layers, indicating that the mutual diffusion had occurred in the Cu and Ni layers. Cu atoms and Ni atoms tend to mutually diffuse and nucleate at the place where the interface energy is the highest [18]. Through the heat treatment, the entire composite layer was strengthened by the formed solid solution alloy, and the interface structure between the Cu and Ni layers was optimized [19]. 

**Figure 5:**
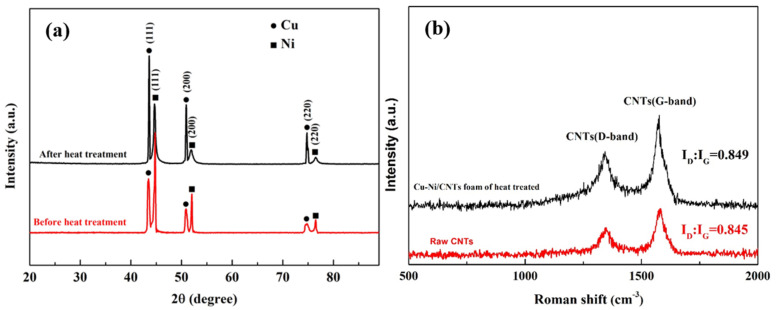
(**a**) The XRD diffraction of Cu-Ni/CNTs foam before and after heat treatment; (**b**) Raman spectra of raw CNTs and Cu-Ni/CNTs foam after heat treatment.

Figure 5a shows the XRD patterns of Cu-Ni/CNTs foams before and after heat treatment. There were both Cu and Ni peaks in the X-ray diffraction pattern before heat treatment. Due to the relatively thin plating layer, X-rays penetrated the plating layer, the XRD patterns also showed the strong diffraction peaks of Cu. The mass fraction of carbon in the foam is measured by a carbon flow analyzer to be 0.25%, too low to be detected by XRD, so there were no diffraction peaks of CNTs observed. After heat treatment, the 2θ values corresponding to Cu (111), (200), (220) crystallographic plane changed from 43.3°, 50.4°, 74.1° to 43.6°, 50.8°, 74.7°, respectively. The slight shift of the diffraction peak to the high angle indicates that a solid solution maybe had formed during the heat treatments, which is consistent with the results reported in the literature [20]. 

Figure 5b shows the Raman spectra of Cu-Ni/CNT foams before and after heat treatment. The D (I_D_ ~ 1336 cm^−1^) and G (I_G_ ~ 1581 cm^−1^) bands were measured to characterize the CNTs disorder degree and the binding mode of the C atom sp^2^. The I_D_/I_G_ value could reflect the integrity degree of the CNT structure [21]. As shown in Figure 5b, the I_D_/I_G_ ratios of the two curves were 0.845 and 0.849, quite similar to each other, indicating almost no damage of the CNT structure during the heat treatments.

**Figure 6:**
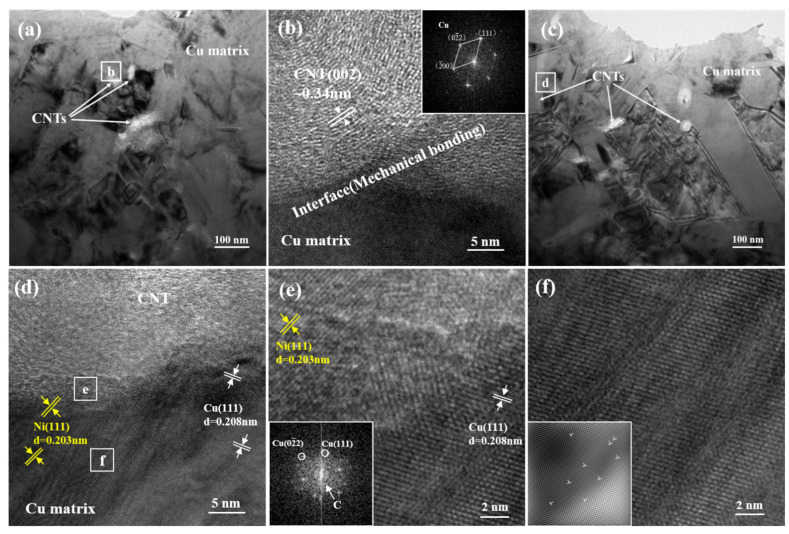
TEM observation of Cu-Ni/CNTs composites before and after heat treatment; (**a**) bright-field image before heat treatment (**b**) enlarged image from the box area in (**b**); (**c**) bright-field image after heat treatment (**d**) enlarged image from the box area in (**c**); (**e**,**f**) are corresponding to the high-resolution images of the marked area in (**d**).

Figure 6 shows the TEM images of Cu-Ni/CNT composites before and after heat treatment. As the bright field images show in Figure 6a,c, the uniformly distributed CNTs were clearly visible in the metal matrix, and the CNTs still maintained a relatively complete tubular structure after electrodeposition and heat treatment. As shown in Figure 6b, the graphite structured CNT and the FCC structured Cu matrix was with a smooth mechanical bonding interface. Because of the poor wettability between Cu and C, this interface is considered to have weak interface bonding force, and the interface is prone to slippage during the load transfer process, thereby reducing the load transfer efficiency [22]. The inserted FFT image in Figure 6b shows that only the Cu matrix was present at the interface and no other oxides or impurities were generated. 

Figure 6d is a further magnified TEM image from the box area in Figure 6c, showing that the CNTs were tightly connected to the metal matrix. An obvious interface transition zone was formed at the interface of Cu and CNTs, and nickel could be found in the copper layer, further confirming the interdiffusion of elements between the inner and outer layers. By performing FFT on the transition zone of the interface, as shown in Figure 6e, the results showed that there was a Cu crystal structure in the transition zone of the interface, and the structure of CNTs became blurred and severely distorted in the transition zone. This may be due to the mutual diffusion of Cu and C atoms during high-temperature heat treatment, which helps to improve the bonding strength between the layers. As shown in area of Figure 6f, some high dislocation density interfacial regions were formed around the CNT/Cu interface. The reason for the formation of these high dislocation density interface regions may be due to the infinite solid solution of the two phases of Cu and Ni.

### 3.2. Compressive Property

**Figure 7:**
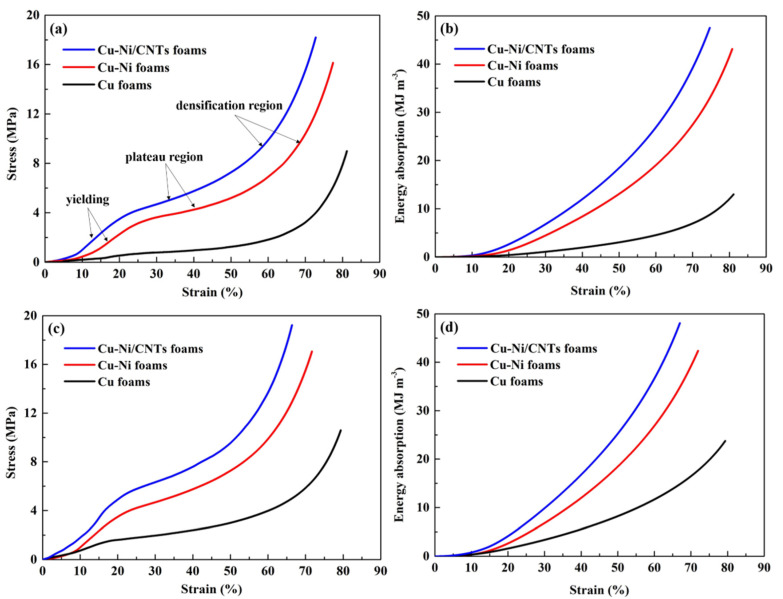
The compressive properties of Cu foams, Cu-Ni foams and Cu-Ni/CNTs foams; (**a**) stress−strain curves before heat treatment; (**b**) energy absorption capacity before heat treatment; (**c**) stress−strain curves after heat treatment; (**d**) energy absorption capacity after heat treatment.

Figure 7a,c show the compressive stress−strain curves of Cu foams, Cu-Ni foams and Cu-Ni/CNTs foams before and after heat treatment. They had the following characteristics: the linear elastic part of the flexible foam was difficult to determine, and there may be slight fluctuations in the platform area that were not absolute levels. Therefore, the deformation of metallic foams can be divided into three regions: a linear region, a similar platform region characterized by a small slope and the densification region characterized by a relatively steep slope in Figure 7a,c [23]. At the same strain, the stress values of Cu-Ni foams increased significantly. Because of the high mechanical properties of the Ni-based coating, the compressive strength of the foams was improved [24]. With the addition of CNTs, the yield strength and the platform-like stress of Cu-Ni/CNTs foams further increased. The overall structure of the CNTs in nickel layer was not destroyed under the heat treatment conditions in this paper, and the heat treatment helped to further improve the interface bonding strength of CNTs and Ni layer. This experimental phenomenon is consistent with References [25,26]. The reason is that the CNTs embedded in the matrix cause a bridging effect during the crack growth process. At the same time, it also ensures load transfer during compression and improves compression performance [27]. 

The specific compressive parameters of heat-treated Cu-Ni/CNTs foams, including the yield strength, plateau stress, and densification strain, are shown in Figure 7c. When the strain was 20%, the stress of Cu-Ni/CNTs foams increased from 3.4 MPa to 4.9 MPa. After heat treatment, the retained good dispersion of CNTs and the solid solution strengthening effect of copper and nickel elements improved the strength of the material. In addition, the diffusion between the Cu and the Ni layers was conducive to improving the bonding force, and the Ni atoms in the Cu layer had good wettability to CNTs, which could be used as a bridge improve interface bonding ability between CNTs and Cu (as shown in Figure 6d), thereby further improving the mechanical properties of composite foams.

Figure 7b,d show the energy absorption capacity (W) of Cu foams, Cu-Ni foams and Cu-Ni/CNTs foams before and after heat treatment. It is defined as the energy per unit volume necessary to deform a given foam material specimen up to a specific strain and can be calculated by

(1) $$W=\int_{0}^{\varepsilon }\sigma d\varepsilon$$

where ε is the compressive strain and σ is the corresponding stress [28]. The area of stress and strain covered by the compression curve is equal to energy absorption, which actually reflects the energy absorption characteristics of the material [29]. It is difficult to determine the sudden change point between the platform area and the dense area of flexible foams [9], the energy absorption value corresponding to the strain of 60% is selected for comparative analysis in this experiment. When the strain reached 60%, the energy absorption of Cu foams, Cu-Ni foams and Cu-Ni/CNTs foams were respectively 4.5 MJ/m^3^, 19.0 MJ/m^3^ and 26.3 MJ/m^3^, as shown in Figure 7b. Obviously, it can significantly improve the compression and energy absorption capacity of Cu foams by electroplating a thin nickel layer. The addition of CNTs increased the energy absorption capacity of Cu-Ni/CNTs foam by 1.38 times, compared with Cu-Ni foam. After heat treatment, the energy absorption of Cu foams, Cu-Ni foams and Cu-Ni/CNTs foams were 10.7 MJ/m^3^, 27.1 MJ/m^3^ and 36.8 MJ/m^3^ as shown in Figure 7d. The best energy absorption ability of 36.8 MJ/m3 was shown by the Cu-Ni/CNTs foams, which was about 39.9% higher than the untreated foams. Therefore, it can be seen that heat treatment can obviously enhance the energy absorption capacity of foams. The heat treatment may make the defect parts of CNTs in the nickel layer form carbides, and strengthen the interface bonding between each other. Chen et al. [30] reported that the preferred position for carbide growth was that CNTs have surface defects with higher surface activity energy. The adhesion was improved between Cu and Ni after heat treatment and the inter-diffusion of Cu and Ni made a good interface bond between Cu and CNTs, which is conducive to the improvement of the energy absorption capacity.

### 3.3. Electromagnetic Shielding Performance

Electromagnetic shielding effectiveness (SE) is the sum of reflection loss (SE_R_), absorption loss (SE_A_) and multiple reflection loss (SE_M_). It can be calculated by the formula Schelkunov [31,32]:

(2) $$\mathrm{SE}={\mathrm{SE}}_{R}+{\mathrm{SE}}_{A}+{\mathrm{SE}}_{M}$$

**Figure 8:**
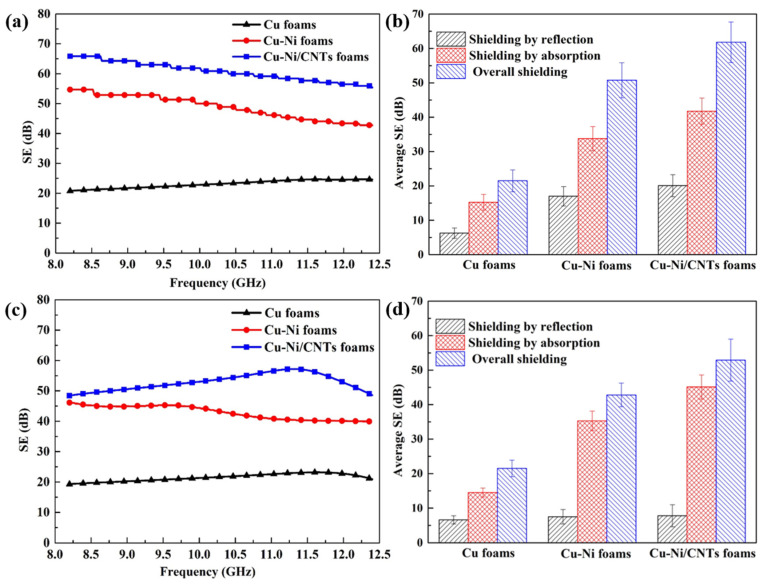
The electromagnetic shielding performance of Cu foams, Cu-Ni foams and Cu-Ni/CNTs foams in the frequency range of 8.2–12.4 GHz; (**a**) SE before heat treatment; (**b**) average EMI SE before heat treatment; (**c**) SE after heat treatment; (**d**) average EMI SE after heat treatment.

Figure 8a shows the changes of SE of Cu foams, Cu-Ni foams and Cu-Ni/CNTs foams in the frequency range of 8.2–12.4 GHz. The maximum values of SE of the Cu foams, the Cu-Ni foams and the Cu-Ni/CNTs foams were 24.66 dB, 55.85 dB, and 67.69 dB, respectively. Figure 8b shows the average SE, SE_R_, and SE_A_ of foams. The average SE_A_, SE_R_, and SE of Cu-Ni/CNTs foams were approximately 20.06 dB, 41.75 dB and 61.81 dB, reflection loss accounted for 32.5%, the absorption loss accounted for 67.5%. It shows that the main shielding mechanism of these foams was absorption loss, which is mainly due to the eddy current loss as the main working principle in the high-frequency electromagnetic field. Compared with the single-layer Cu foams, the electromagnetic shielding performance of the double-layer Cu-Ni foams was improved by about 2.26 times. The main reason is that the addition of nickel layer increased the thickness of the foam skeleton, which is conducive to the improvement of electromagnetic shielding performance. In addition, the multilayer structure increases the number and probability of reflecting and absorbing electromagnetic waves due to impedance mismatch [15]. The nickel layer can make the electromagnetic waves reflected by the copper layer re-enter the nickel layer to be absorbed, further increasing the material inside multiple reflection and scattering loss of electromagnetic waves [33,34,35]. For Cu-Ni/CNTs foams, the average electromagnetic shielding effect showed the best value of 61.81 dB. The main reason is that CNTs have higher electrical conductivity and higher node loss tangent, which can rely on the electronic polarization and interface polarization of the medium to produce reflection loss. In addition, CNTs with small size effects are prone to produce interface polarization and multiple scattering inside the material, so that electromagnetic waves are fully reflected and absorbed in the metal skeleton. The addition of CNTs makes CNT/Cu-Ni composite foam have more impedance mismatch interfaces than Cu-Ni foam, and improves the reflection coefficient of the material to electromagnetic waves, so its electromagnetic shielding effectiveness is higher. 

Figure 8c shows the changing trend of the electromagnetic shielding of samples after heat treatment. The experimental results show that the average SE of the single-layer Cu foams hardly changed, the average SE of the double-layer Cu-Ni foams reduced from 50.75 to 43.35 dB, and the average SE of the Cu-Ni/CNTs foams reduced from 61.81 dB to 52.9 dB. As shown in Figure 8d, the contribution of the absorption loss of the three foam materials was significantly higher than the reflection loss, which was still the main electromagnetic shielding mechanism. The heat treatment caused a slight reduction of the electromagnetic shielding performance of the double-layer foams. The interdiffusion of Cu and Ni destroyed the periodicity of the lattice arrangement of metal crystals and increased lattice distortion. Generally, the more severe the lattice distortion of the crystal, the greater the obstacle to the movement of electrons, and the smaller the electrical conductivity of the material [36]. In addition, there are grain boundaries, twins, dislocations, etc., in the crystal structure after heat treatment (as shown in Figure 6c,f). These microscopic crystal structures also affect the electromagnetic properties of the materials, resulting in differences in the electromagnetic shielding performance of the materials.

To further highlight the merit of CNTs/Cu-Ni foams, the shielding performance of different composites is listed in Table 2. As shown in the table, the composite foams obtained before and after heat treatment exhibited excellent electromagnetic interference shielding performance mainly based on absorption. This work provides the possibility for the further application of high-performance electromagnetic interference shielding materials in the fields of construction, aerospace and electronics.

## 4. Conclusions

The composite foams with a homogeneous dispersion of embedded CNTs in the inner Cu layer and outer Ni layer were prepared through chemical plating and electrodeposition. A significant improvement in the compressive strength, energy absorption and electromagnetic shielding performance was shown. The subsequent heat treatment led to the interdiffusion of the Ni and Cu layers and CNT/Cu interface bonding improvement, and resulted in an increase of the compressive strength and a slight decrease of the SE. whether before or after heat treatments, the absorption loss was the main electromagnetic shielding mechanism, based on the composite component and foam structure.

## Figures and Tables

**Table 1:** The sample name and related parameters.

Sample	Density (g cm^−3^)	Porosity (%)
Cu foams	0.98	89.0
Cu-Ni foams	1.13	87.3
Cu-Ni/CNTs foams	1.14	87.1

**Table 2:** The shielding performance comparison of different composites.

Materials	Method	Frequency (GHz)	SE(dB)	Ref.
Cu-Ni/CNTs foam (before heat treatment)	electrodeposition	8.2–12.4	61.8	This work
Cu-Ni/CNTs foam (after heat treatment)	electrodeposition	8.2–12.4	52.9	This work
Cu-Ni-CNTs foam	electroless plating and electrophoretic deposition	8.2–12.4	47.5	[15]
CNTs/Cu foam	electrodeposition	8.2–12.4	33.6	[16]
open cell Al-foam	replica impregnation method	8.2–12.4	44.6	[37]
Ni/Cu/metallic glass/Cu/Ni composite	electroless plating	8–12	35	[38]
PVDF/Ni-chains composite foam	compression molding	8.2–12.4	26.8	[39]
CNT/TPU	microwave selective sintering	8–18	34	[40]

## Data Availability

The data presented in this study are available on request from the corresponding author.
